# The decision to purchase genome edited food products by Iranian consumers: theory of planned behavior as a social intervention tool

**DOI:** 10.3389/fgeed.2025.1483510

**Published:** 2025-09-04

**Authors:** Naser Valizadeh, Shobeir Karami

**Affiliations:** ^1^ Department of Agricultural Extension and Education, School of Agriculture, Shiraz University, Shiraz, Iran; ^2^ Persian Gulf Research Institute, Persian Gulf University, Bushehr, Iran

**Keywords:** consumers’ preference, willingness to buy, attitude towards gene-edited products, trust in gene-edited products, behavioral interventions

## Abstract

The main aim of present study was to analyze the consumers’ preferences about genome/gene-edited food products in Iran. For this purpose, an extended version of the theory of planned behavior was used as a social intervention tool. The theory of planned behavior was firstly extended using the introduction and new variable of trust in gene-edited products and perceived benefits of gene-edited food products, but in the next step, it was also analyzed statistically. To achieve the main objective of the research, a representative sample was selected from the population of purchasers of gene-edited products, and data were collected using a cross-sectional survey. The validity and reliability of the data collection tool was evaluated and confirmed using different quantitative and qualitative methods in the pilot stages and after the main survey. The results of structural equation modeling showed that the attitude towards gene-edited food products, perceived behavioral control, and the subjective norms of gene-edited products had positive and significant effects on the intention to purchase these products. The results of the study indicated that two newly introduced variables to the theory of planned behavior, namely, trust in gene-edited products and the perceived benefits of gene-edited products also had positive and significant effects on the intention to purchase these products. Based on the results, the framework employed and extended in this study can provide the basis for effective interventions to improve consumers’ preference for gene-edited food products. Also, some practical suggestions were provided for policymakers, managers, and producers of these products.

## 1 Introduction

The world today must increase food production to meet the demands of a growing population. However, this could also lead to the end of the human era on the planet (Dima et al., 2023). Therefore, humans must use their maximum power to escape from this created food insecurity, and to continue the life of their species (Ewa et al., 2022). If the severity of food insecurity is not the same in the world and is mixed with other types of insecurity, such as economic insecurity and biological insecurity, migration to safer areas of the world will be one of the first expected consequences (Nogue et al., 2024). Therefore, examining ways out of this situation will be one of the most important priorities of decision-makers in different regions of the world.

Addressing food insecurity needs multidimensional and comprehensive strategies that encompass political, social, and technological domains (Righettini and Bordin, 2023). Among these, scientific and technological advances in agriculture have been increasingly emphasized for their potential for long-term sustainability (Getahun et al., 2024). One of the new innovations is genome editing, particularly in the field of crop improvement (Jouanin et al., 2018; Nogue et al., 2024). Genome editing is an innovative method to enable scientists to make precise, targeted alterations to the DNA of organisms, including crop plants. Unlike traditional breeding or even earlier forms of genetic modification, genome editing techniques (e.g., CRISPR-Cas9) do not necessarily offer inserted foreign DNA, which is likely to provide more acceptance by consumers and regulatory bodies (Nkott and Temple, 2021; Borrello et al., 2021; Zhang et al., 2024).

Genome editing offers some potentially appealing characteristics. These characteristics could increase crop resistance to drought and pests, improve nutritional quality, lower production costs, and enhance yield efficiency with climate stress (Araki and Ishii, 2015; Bearth et al., 2024). Therefore, many nations have dedicated funds to the development and commercialization of genome edited food products (Menz et al., 2020). Furthermore, the influence of genome editing in practice will be unquestionably influenced by consumer acceptance, that is central to the market demand (Uddin et al., 2022; Bearth et al., 2024).

The introduction of genome edited foods to international and regional markets has stimulated continuing debates on ethical, legality and consumer issues (Eriksson et al., 2018; Nkott and Temple, 2021). Much of the debate focuses on issues around the transparency of labelling, risks to health, potential risks to the environment, corporate ownership of technologies, and unknowns in terms of future developments or consequences of gene editing (Araki and Ishii, 2015; Tabei et al., 2020; Spok et al., 2022; Macall et al., 2023). Despite the scientific advancements, regulatory frameworks in many countries are still evolving, and public awareness and understanding of genome editing remain limited (Ewa et al., 2022; Hendricks et al., 2022). In addition, consumer perceptions and attitudes can be significant factors in consumer acceptance and the commercial uptake of genome edited foods (Araki and Ishii, 2015). Products of genome editing often carry more ambiguous public attitudes than genetically modified organisms (GMOs), partly due to the perceptions of ‘naturalness’ and targeted improvements (Britton and Tonsor, 2020; Selfa et al., 2021). Public attitudes toward genome editing products can be commonly resistant mainly due to distrust in science and scientists, lack of knowledge and understanding, and ethics (Nkott and Temple, 2021).

Nonetheless, the current literature on consumer attitudes towards genome-edited food is predominantly focused on developed contexts (Gao et al., 2024), which raises a significant gap in our understanding of consumer behavior in developing contexts like Iran (Akbari et al., 2023). In the case of Iran, just as with many developing contexts, food security, consumer safety and sustainable agriculture are all primary national priorities (Valizadeh and Hayati, 2021). In this context, we do not know the specific effect of psychological and cultural factors influencing Iranian consumers on decisions regarding genome-edited foods. This context-specific understanding of consumer behavior presents a significant research gap, particularly when trust, perceptions of benefits, and familiarity with new food technologies vary across regions. In addition, most of the existing studies generalize genome-edited foods as simply GMOs instead of understanding the distinct technical attributes and consumer considerations. In this case, a more customized and empirical approach is required to understand how consumers in different socio-economic contexts and regulatory environments, respond to these new technologies. Accordingly, this study seeks to explore the decision process of Iranian consumers when selecting labelled genome edited food products. This investigation emphasizes not only attitudes and perceptions but the psychological process of consumers’ decisions in the context of both food choice and technological innovation. The key research question that underlies this study is: What factors influence Iranian consumers’ intention to purchase genome edited food products in the presence of labels?

To answer this research question, the study deploys an extended model of Theory of Planned Behavior (TPB). TPB is a well-established social-psychological theory used in research as a framework to understand human decision making. Typically, TPB considers three constructs: attitudes, subjective norms, and perceived behavioral control. However, the study added several other knowledge-related factors applicable to the context, including trust and perceived benefits. By extending TPB to include these knowledge-related and trust-based constructs, the study offers a more comprehensive view of consumer behavior in the Iranian context. The added constructs provided a more complete picture of the behavioral intentions. The key novelty of this study is that it applied an extended TPB model to a less-studied population (consumers in Iran) and specifically to labeled genome edited food and food products rather than GMOs as a class. The socio-cultural specificity of food perception and consumption warrants our understanding of ordering and driving that behavior as it is important for both domestic policy and international discussions on genome editing. The study has both theoretical implications and practical implications for policymakers, industry, and science communicators. Study findings could help policymakers and industry design educational materials, develop labeling policies that better articulate consumer interests, and developing regulation in ways that reflect public values and allows for innovation. Study findings on the behavior underpinning acceptance of genome edited products may also capture lessons on resilience, guiding future market strategies, and risk communications.

The remainder of this paper is organized as follows: Section 2 provides a review of the relevant literature on consumer behavior and genome edited food. Section 3 outlines the theoretical framework and hypotheses. Section 4 describes the methodology, including sampling and data analysis procedures. Section 5 presents the results. Section 6 discusses the findings in light of existing research and implications. Finally, Section 7 concludes with recommendations and future research directions.

## 2 Theoretical background

### 2.1 Theory of planned behavior

The theory of planned behavior (TPB) was proposed for the first time in 1985 (Moon, 2021) and is considered the most famous theory in explaining the tendencies of different groups of people, especially consumers. This theory originates from Ajzen and Fishbein’s theory of Reasoned Action, which was developed to respond to the lack of correspondence between people’s general tendencies (Conner, 2020). The TPB focuses on behavior and looks for the effect of other variables such as social (subjective) norms and belief in individual self-efficacy on behavior. According to the TPB, human action is affected by three major factors: positive or negative evaluation of behavior (attitude towards behavior), understanding of social norms resulting from performing or not performing behavior (subjective norms) and understanding of the capacity to perform behavior (self-efficacy or understanding of perceived behavioral control) (Savari and Khaleghi, 2023; Savari et al., 2023). By combining attitude towards behavior, subjective norms and perceived behavioral control, intention to behavior is obtained. Having a strong attitude and subjective norm towards the behavior and perceived behavioral control, the individual’s intention to perform the behavior increases (Ajzen, 2020).

### 2.2 Hypothesis development

#### 2.2.1 The effect of attitude on intention

Rarely can one find a more interesting subject than attitude in the study of a society, because by measuring attitude one can provide a guide to a complex world. There is a general agreement that attitude is acquired and not inherent. Society is like a mosaic with heterogeneous parts, but attitude is always the main component in its design (Ates, 2020). Attitude is important because it affects people’s understanding of their physical and social world and affects their actual behavior. When there is a discussion about measuring attitude, attitude means the relationship between the object of attitude and the evaluated classes (such as good and bad) (Conner, 2020). Attitude has judgmental and memory components. The memory component includes presenting the attitude in permanent memory. The judgmental component includes a direct evaluation originating from memory about an issue at a specific time and place. Memory is the basis of judgment and attitude about a subject (Tama et al., 2021). As it is now used in psychology, the term attitude refers to a hypothetical variable, which is a basis for evaluating some issues in a favorable or unfavorable behavior (Ajzen, 2020; Savari et al., 2023). This background cannot be observed directly and it must be inferred from people’s responses to the topic of attitude measurement. This conclusion can be obtained from actual behavior (such as going towards or avoiding the measured subject) and clear verbal statements (answers to attitude measurement questions) (Ulker-Demirel and Ciftci, 2020). Attitude includes feelings, beliefs, and positive and negative orientation about different subjects. This is a summarized feature of attitude that makes it efficient, flexible, and adaptable (Savari and Cgharechaee, 2020). Theorists such as Ajzen and Fishbein in 1975 limited attitude in cognitive processes and stated that in order to determine the development of attitude, cognition structures should be examined (Conner, 2020). This point of view has been challenged by many experts who have been looking for evidence to support the influencing factors on attitudinal foundations and have shown that attitude can be influenced by many natural stimuli (Coskun and Yetkin Ozbuk, 2020). Examining the relationship between respondents’ attitudes towards gene-edited food products and their intention to purchase and consume these products is one of the hypotheses that was analyzed in this research. The review of existing studies in this field showed that this relationship is a positive relationship (Bearth et al., 2024; Bechtold, 2018; Macall et al., 2023; McFadden et al., 2023; Ploll et al., 2023; Sleboda and Lagerkvist, 2022; Ufer et al., 2019; Ufer et al., 2022). Based on this, it is hypothesized in this study that attitude has a positive and significant effect on intention.

#### 2.2.2 The effect of subjective norms on intention

Ajzen (2020) proposes that subjective norms are how an individual understands the social pressures or expectations from significant others about whether they should do, or not do, a certain behavior. Subjective norms reflect the internalized knowledge of social rules and expectations individuals develop that shape behavioral intentions. The empirical literature supports a positive relationship between subjective norms and behavioral intentions, across all non-health behavior domains (Cummings and Peters, 2022; Matsuo and Tachikawa, 2022; Nkott and Temple, 2021; Ploll et al., 2023; Partonezhad et al., 2025). Therefore, when individuals feel more social pressure or encouragement from their family, friends, or community for doing a behavior as described in this study, their intention in this study to do that behavior increases significantly. In the example of consumers evaluating gene-edited food products, social influence can be essential, since gene-edited food products are often novel and can be controversial. Therefore, it is helpful to understand how respondents’ subjective norms shaped their intentions to inform consumer behaviors. With that in mind, examining the influence of respondents’ subjective norms on their intention to purchase and consume gene-edited food products was identified as a primary objective of the present study, to capture the social aspect of consumer behavior in this emerging market.

#### 2.2.3 The effect of perceived behavioral control on intention

The third core construct within the Theory of Planned Behavior is the relationship between perceived behavioral control and intention. Perceived behavioral control relates to an individual’s perception of their ability to perform a given behavior, which includes aspects like ease or difficulty, and self-efficacy (Ataei et al., 2021a). The literature shows that perceived behavioral control positively and significantly impacts consumers intentions to purchase gene-edited food products (Araki and Ishii, 2015; Bechtold, 2018; Ataei et al., 2021b; Spok et al., 2022). When consumers are confident in their ability to access and use gene-edited foods, their intentions to purchase gene-edit products increase. This leads to the study of the relationship between perceived behavioral control and intentions to purchase gene-edited food products, being the third hypothesis of the current study. An important distinction to mention within the Theory of Planned Behavior is the difference between perceived behavioral control and actual control (Ulker-Demirel and Ciftci, 2020). Perceived behavioral control is an individual’s subjective evaluation of his/her ability and confidence to do a behavior, with the assessment taking into consideration accessibility, resources and self-efficacy, while actual control is considered objective and external including legal regulations, availability in the market, economic limitations, and social circumstances that constrain or enable the behavior. While perceived behavioral control may reflect actual control to some degree, perceived and actual control are not synonymous. For example, a consumer might have confidence in purchasing gene-edited food products, yet have barriers to actual behavior from limited accessibility to products or excessive pricing. Despite the disparities between perceived and actual control, perceived behavioral control is still an important predictor of behavioral intention, as it establishes an individual’s belief in their ability to act. Because of the defining role of perceived behavioral control in enabling or constraining consumer behavior, it stands to reason that as perceived behavioral control increases, so too would the increase of a positive affectation to consumer’s intention to purchase gene-edited food products.

#### 2.2.4 The effect of trust on intention

Consumer trust in gene-edited food products plays an important and complex role in influencing intention to purchase these new products. Many studies, including Cummings and Peters (2022), Araki and Ishii (2015), Bearth et al. (2024), Britton and Tonsor (2020), Hendricks et al. (2022), and Ufer et al. (2022) found that a greater trust led to a stronger intention to purchase gene-edited foods. Trust encompasses beliefs about safety, ethical standards, transparency, and reliability - which are especially salient in light of the newness and complexity of gene editing technology. Consumers who have confidence in regulatory oversight, that scientific evidence supports these products, and that the products themselves provide benefits will develop and more steadfast intention to purchase. Furthermore, if the consumer does not trust the products in any of those areas, they may not be as confident in their intentions, or they may maintain some skepticism or reluctance. In short, consumer trust is the linchpin to casual actions that lead consumers to consume gene-edited food. Thus, developing and preserving consumer trust is necessary to support positive purchase intentions and total market benefits of gene-edited food products.

#### 2.2.5 The effect of trust on attitude

Trust also has a strong influence on the attitudes of consumers toward gene-edited food products by impacting the become favorable or unfavorable attitudes toward the foods. Trust can link with more favorable attitudes, as studies by Cummings and Peters (2022), Araki and Ishii (2015), Bearth et al. (2024), Britton and Tonsor (2020), Hendricks et al. (2022), Muringai et al. (2020), Ufer et al. (2019), and Ufer et al. (2022) have concluded that there is a strong relationship with increased levels of trust and more favorable attitudes. Trust influences more favorable overall evaluations as consumers believe gene-edited products are safe, ethically produced, and beneficial. The supportive attitude toward gene-edited food products is significant as attitude is a gateway to acceptance related to willingness to use food technology. Trust is a strong mechanism to reduce perceived risks and uncertainties that may be present when adopting novel products, as trust can create a consumer who is comfortable and open-minded, fostering a supportive nature to gene-edited foods and increasing the likelihood of support and recommendations. Thus by having strong trust-building communication, or endorsement through balanced knowledge and credible organization, attitudes toward gene editing could be positively impacted.

#### 2.2.6 The effect of perceived benefits from GE foods on intention

Consumers’ intentions to purchase gene-edited foods are directly and positively influenced by perceived benefits of gene-edited foods. Studies conducted by Bearth et al. (2024), Macall et al. (2023) and McFadden et al. (2023) highlight that as consumers become informed about the perceived benefits of gene-edited foods, such as nutrition, environmental benefits, and food safety, their intentions to purchase gene-edited foods increases. Furthermore, understanding perceived benefits, which alleviate fears surrounding unknown technologies, can motivate consumers to choose gene-edited food products over conventional products. Perceived benefits, are typically viewed as a rational justification for a particular purchasing decision. They also serve as emotional reassurance, ensuring that their food choice contributes to positive social and environmental outcomes. Perceived benefits can also reinforce education and marketing efforts to accentuate their motivations, making them feel more compelled to adopt gene-edited products in the marketplace.

#### 2.2.7 The effect of perceived benefits from GE foods on trust

Perceived benefits of gene-edited foods are also a critical factor in strengthening consumer trust in these foods. Several authors (Araki and Ishii, 2015; Bearth et al., 2024; Eriksson et al., 2018; Hendricks et al., 2022; Macall et al., 2023; Muringai et al., 2020; Spok et al., 2022) find that when consumers see clear advantages, such as health benefits, environmental benefits, or ethical benefits, their trust in gene-edited foods’ safety and dependability is enhanced. This increasing trust reduces scepticism and increases acceptance by providing assurance to consumers that the products are beneficial and safe to eat. In many cases, perceived advantages are perceived as solid evidence that counteracts the fears or misrepresentation that is sometimes fed to consumers through media or social media. Therefore, improving awareness and understanding of these benefits can enhance consumer confidence and trust that are very important for long-term acceptance and successful market entry of gene-edited foods.

#### 2.2.8 The effect of perceived benefits from GE foods on attitude

The perceived benefits of gene-editing foods have a strong positive influence on consumers’ attitudes toward gene-edited foods. There is considerable research evidence from Hendricks et al. (2022), Macall et al. (2023), McFadden et al. (2023), Muringai et al. (2020), and Sleboda and LagerKvist (2022) that show how consumers shift their attitudes to be more positive when their perceptions of benefits, like food safety, nutritional quality, and environmental sustainability, become stronger. Positive attitudes are vital because they represent the antecedents to both intention and behavior. Consumers who accept those benefits will experience, at least relatively, a more positive, nonresistant and open attitude toward gene-edited foods. Additionally, positive attitudes moderated by perceived benefits are also good for word-of-mouth recommendations and social acceptance in the community, enhancing the effect of perceived benefits. Thus, in reference to attitude development, it is essential to communicate and demonstrate the benefits from gene editing to foster consumer attitudes and build acceptance of gene-edited food products.

**FIGURE 1:**
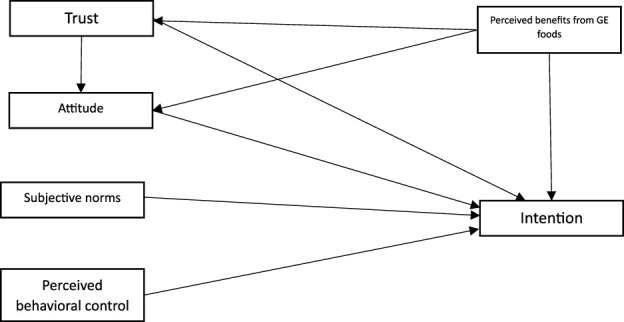
Extended version of TPB.

Finally, the extended version of the TPB to analyze consumers’ intention to purchase gene-edited foods products is presented in Figure 1.

## 3 Research methods

### 3.1 Development of the initial draft of the research tool

The study questionnaire was developed in Farsi and two main processes were carried out on it. In the first stage, questionnaire questions were extracted using the literature review and research background in the field of gene-edited food products. For this purpose, keywords such as socio-psychological determinants of consumers’ behavior, intention to buy gene-edited food products, theory of planned behavior, measurement of socio-psychological variables, etc. were used. Then the items and variables used in these studies were compared with each other to select the best and most relevant items to measure the variables. In the second step, the think-aloud technique was used. According to Zhang et al. (2017), this technique allows researchers to collect more information. Think-aloud technique allows the respondents to think and give feedback on the answers they give to the questions. This technique also helps the respondents to discuss their thoughts on the questions. This process allows the respondents to have enough time to analyze and consider the answers and options against their life experiences. Clarity and language and phrasing were also examined at this stage. This work was done using six experts. The professionals consulted for content validity were academics and practitioners with expertise in agricultural biotechnology, consumer behavior, and food science. They provided content validity expertise that ensured the questionnaire was scientifically valid as well as relevant in context. These two stages led to the verification of the face and content validity of the questionnaire by making changes in the questions and removing/adding some items.

After verifying the face and content validity, 30 questionnaires were filled among buyers of gene-edited food products in Shiraz city. For this purpose, the researchers randomly selected one of the hypermarkets in Shiraz city in Iran as the place to conduct the pilot test and filled 30 questionnaires among the Buyers. The results of this part of the study were used to calculate Cronbach’s alpha, which is one of the reliability indicators of the questionnaire. The calculation of Cronbach’s alpha coefficients for the research variables showed that the values of all these coefficients are at an acceptable level. At this stage, the questionnaire was prepared for the main survey of buyers of gene-edited food products.

### 3.2 Statistical population and sampling method

The statistical population of this study was the buyers of genome edited foods in the city of Shiraz, Iran. Shiraz was chosen as the data collection site due to its diverse demographic profile, urban consumer behavior, and representative socio-cultural characteristics relative to a larger Iranian urban population. Furthermore, Shiraz was a realistic and sensible choice given logistical feasibility, and our budget limitations. Considering that the size of the population was not known in this study, the statistical power method was used to estimate the sample size. Therefore, the sample size was extracted using Cohen’s tables (Cohen, 1992) for the Pearson correlation test. Small to medium effect size, statistical power, and alpha used for this purpose were equal to 0.214, 0.05 and 0.80, respectively. Using this method, the sample size was estimated to be 234 cases. For this purpose, first a list of Shiraz hypermarkets was prepared. They were then asked to indicate whether or not they sell gene-edited food products. Some of the hypermarkets stated that they do not sell gene-edited food products. But most of them sold these products if they have labels. Finally, six hypermarkets in different areas of Shiraz city were selected as the main place of data collection from the respondents. To select the samples, the data collectors appeared in the hypermarkets at certain hours and explained the objectives of the work to each of the buyers and asked them to answer the questionnaire if they wanted to.

### 3.3 Questionnaire structure and operational definitions of variables

After providing informed consent, participants were asked to answer the questionnaire. This questionnaire was presented to the respondents in the form of three main sections. In the first part of the questionnaire, the purpose of the work was explained to respondents and also a definition of genome edited food products was presented to them. In the next step, questions were asked about their literacy level and their personal and professional characteristics. In the third part, the questions used to measure each of the underlying variables such as intention to purchase genome edited food products, perceived benefits from GE foods, trust in genome edited food products, attitude towards genome edited food products, subjective norms of genome edited food products, and perceived behavioral control about genome edited food products were presented. Intention to purchase genome edited food products in this study was measured using four items. These items were “if the genome edited food product is available in the hypermarket, I tend to buy it,” “I recommend buying genome edited food products to others,” “if I cannot find the genome edited food products in this hypermarket, I will go to other hypermarkets to buy them,” “I am willing to buy genome edited food products even if they are banned later.” These items were extracted by making changes from the studies of Chen (2008) and Valizadeh and Hayati (2021).

Perceived benefits from GE foods were measured using three items including “gene-edited products have a higher nutritional value than similar non-edited products,” “gene-edited products have relatively lower prices than similar products,” and “gene-edited products are not dangerous for human health.” These items were also adapted with changes from studies of Lassoued et al. (2019). Trust in genome edited food products was the next factor that was measured indirectly (in the form of latent variable) using three items (1- I trust genome edited food products because I’m sure scientists have done enough research on their health, 2- I trust genome edited food products because I think the food industry has strict standards, and 3- I trust genome edited food products because I know that decision-makers and statesmen are also confident in their health).

The three main variables in the original version of the theory of planned behavior included attitude towards genome edited food products, subjective norms of genome edited food products, and perceived behavioral control about genome edited food products. Four items “the use of gene editing for food production is very good,” “the use of gene editing for food production is very wise,” “I completely agree with the use of gene editing technology for food production,” and “I do not think that the use of gene editing to produce food products a risk to environment, myself, and other people” were used to measure attitude towards genome edited food products. These items were used in study of Chen (2008) and the authors used them by making changes to match them with this study. Measurement of subjective norms of genome edited food products was also done using three items. These items which included “my family encourages me to buy genome edited food products,” “experts who influence me think that I should buy genome edited food products,” and “my friends encourage me to buy genome edited food products” were used in studies of Chen (2008) and Akbari et al. (2019) and they were used by applying changes in them according to the goals of the current research. The last latent variable studied was perceived behavioral control about genome edited food products, which was measured using four items “whether or not I end up buying genome edited food products is entirely up to me,” “if there are genome edited food products in the hypermarket, nothing prevents me from buying them,” “I can easily buy genome edited food products whenever I want,” and “I think that all consumers have easy access to genome edited food products “which were adapted from the studies of Chen (2008) and Valizadeh et al. (2023). It should also be mentioned that all constructs were measured using a five-point Likert scale, ranging from 1 (Completely Disagree) to 5 (Completely Agree).

### 3.4 Variable reliability and validity, discriminant validity, and collinearity statistics

Cronbach’s alpha, Rho-A, composite reliability, and average variance extracted (AVE) for variable reliability and validity analysis of latent variables of intention to purchase genome edited food products, perceived benefits from GE foods, trust in genome edited food products, attitude towards genome edited food products, subjective norm of genome edited food products, and perceived behavioral control about genome edited food products were used. In order to verify variable reliability and validity, Cronbach’s alpha, composite reliability and Rho-A values must be greater than or equal to 0.7. But the value of AVE must be greater than or equal to 0.5. These values will be analyzed in the results section. Discriminant validity was evaluated using Fornell-Larcker statistics and cross loadings. In order to check the collinearity between variables and items, inner variance inflation factor and outer variance inflation factor were used, respectively.

### 3.5 Method of analyzing data and results

Excel, SPSS26 and SMARTpls3 software packages were used for data analysis. But it should be noted that the main analysis of this study is based on structural equation modeling using SMARTpls3.

## 4 Results

The evaluation of the structural model of the research using path coefficients showed that the perceived behavioral control in the field of gene-edited products had the greatest effect on the intention to purchase gene-edited products (Table 1). It is also necessary to mention the effect this variable on the willingness to buy gene-edited products was positive (beta value 0.370). The second influential variable on the intention to buy gene-edited products was attitude. This variable with a beta value of 0.301 had a significant effect on explaining the intention to buy gene-edited products. The two variables perceived benefits from GE foods and subjective norms influenced the intention to buy gene-edited products in a positive and significant way. Beta values of perceived benefits from GE foods and subjective norms were 0.181 and 0.153 respectively (Table 1). In this study, there were two mediating parameters (variables), some variables had direct effects on them. These two variables were attitude towards gene-edited products and trust in gene-edited products. The analysis of path coefficients showed that perceived benefits from GE foods with a beta value of 0.416 has the greatest effect on the attitude towards gene-edited products. Also, the trust with a beta equal to 0.138 was the second variable that had a significant effect on the attitude towards gene-edited products. The analysis of direct effects on the variable of trust towards gene-edited products showed that perceived benefits from GE foods with a beta equal to 0.527 had a powerful significant effect on the explanation of its dependent variable.

**TABLE 1:** Path Coefficients of the theoretical framework.

Variable	Attitude	Intention	Perceived behavioral control	Perceived benefits from GE foods	Subjective norms	Trust
Attitude		0.301				
Intention						
Perceived behavioral control		0.370				
Perceived benefits from GE foods	0.416	0.181				0.527
Subjective norms		0.153				
Trust	0.138					

The analysis of total indirect effects on the dependent variables showed that among the variables that have an indirect effect on the intention to buy gene-edited products, perceived benefits from GE foods had the largest indirect effect (0.147) (Table 2). However, the indirect effect of trust in gene-edited products on the intention to buy gene-edited products was not significant (the indirect effect was equal to 0.041). Also, perceived benefits from GE foods had a significant effect of equal to 0.073 on the attitude towards gene-edited products (Table 2).

**TABLE 2:** Total Indirect Effects of independent variables on the dependent variables.

Variable	Attitude	Intention	Perceived behavioral control	Perceived benefits from GE foods	Subjective norms	Trust
Attitude						
Intention						
Perceived behavioral control						
Perceived benefits from GE foods	0.073	0.147				
Subjective norms						
Trust		0.041				

The analysis of the specific indirect effects of each of the independent variables on the intention to buy gene-edited products showed that perceived benefits from GE foods has an effect on it from two ways (Table 3). Examining these two paths revealed that the path “perceived benefits from GE foods -> Attitude -> Intention” had a stronger effect than the path “perceived benefits from GE foods -> Trust -> Attitude -> Intention.” In other words, the first path accounts for a larger share of the total indirect effect reported in the previous section. The beta values of these two paths were 0.125 and 0.022, respectively. The values of the specific indirect effects of perceived benefits from GE foods and trust on the intention to buy gene-edited products were equal to the values of their overall effects (Table 3).

**TABLE 3:** Specific indirect effects.

Hypothesis	Specific indirect effects
Perceived benefits from GE foods -> Trust -> Attitude	0.073
Perceived benefits from GE foods -> Attitude -> Intention	0.125
Trust -> Attitude -> Intention	0.041
Perceived benefits from GE foods -> Trust -> Attitude -> Intention	0.022

The direct effects of independent variables on the intention to buy gene-edited products are obtained from the sum of direct and indirect effects. Table 4 shows the total effects of each of the variables in the conceptual framework. Examining these effects showed that among the influencing variables on the intention to buy gene-edited products, perceived behavioral control has the greatest effect (beta value was 0.370). The second variable with the largest indirect effect on the intention to buy gene-edited products was perceived benefits from GE foods, which had a beta of 0.328. Also, the attitude towards gene-edited products had a significant total effect of 0.301 on the intention to buy gene-edited products. The two variables of subjective norms and trust had total effects of 0.153 and 0.041 respectively on the intention to buy gene-edited products. The total effect value of perceived benefits from GE foods and trust on the attitude towards genetically modified products was equal to 0.488 and 0.138, respectively. The total effect of perceived benefits from GE foods on trust in gene-edited products was equal to 0.527, which was the highest total effect among all the variables in the research framework (Table 4).

**TABLE 4:** Total effects of the independent variables on dependent variables.

Variable	Attitude	Intention	Perceived behavioral control	Perceived benefits from GE foods	Subjective norms	Trust
Attitude		0.301				
Intention						
Perceived behavioral control		0.370				
Perceived benefits from GE foods	0.488	0.328				0.527
Subjective norms		0.153				
Trust	0.138	0.041				

This part of the analysis was related to the measurement model of consumers’ intention towards gene-edited products, in which the relationship between each of the latent variables and items measuring them was examined. To measure this relationship, the outer loadings index was first used, which actually shows the degree of correlation of each item with its corresponding variables (Table 5). For example, the first item of attitude has an outer loading equal to 0.843 with the variable of attitude towards gene-edited products. This shows that the correlation of this item with the attitude towards gene-edited products is at a high level. Therefore, more likely, this item belongs to the underlying variable of attitude.

**TABLE 5:** Outer loadings of items in measurement model.

Item/index	Attitude		Intention		Perceived behavioral control		Perceived benefits from GE foods		Subjective norms		Trust	
	Loading	Weights	Loading	Weights	Loading	Weights	Loading	Weights	Loading	Weights	Loading	Weights
Attitude 1	0.843	0.360										
Attitude 2	0.879	0.271										
Attitude 3	0.808	0.231										
Attitude 4	0.859	0.317										
Intention 1			0.836	0.318								
Intention 2			0.857	0.305								
Intention 3			0.758	0.272								
Intention 4			0.863	0.309								
PBC 1					0.764	0.354						
PBC 2					0.776	0.294						
PBC 3					0.748	0.280						
PBC 4					0.804	0.364						
PBGEF 1							0.747	0.399				
PBGEF 2							0.872	0.484				
PBGEF 3							0.773	0.361				
Subjective Norm 1									0.888	0.413		
Subjective Norm 2									0.857	0.337		
Subjective Norm 3									0.842	0.410		
Trust 1											0.799	0.418
Trust 2											0.773	0.310
Trust 3											0.864	0.494
Cronbach’s Alpha	0.871		0.848		0.777		0.716		0.828		0.748	
rho_A	0.889		0.852		0.783		0.737		0.835		0776	
CR	0.911		0.898		0.856		0.841		0.897		0.854	
AVE	0.718		0.688		0.598		0.639		0.744		0.661	

Table 5 shows the outer loadings of each of the variable with their corresponding variables. The necessary cut-off point for this statistic is 0.7. In other words, if the outer loading value of an item with its corresponding latent variable is greater than or equal to 0.7, that item is accepted. As the results of Table 5 show, all the items measuring attitude towards gene-edited products, intention to purchase gene-edited products, perceived behavioral control, subjective norm of gene-edited products, trust in gene-edited products, and perceived benefits from GE foods had acceptable outer loading values (Table 5).

**FIGURE 2:**
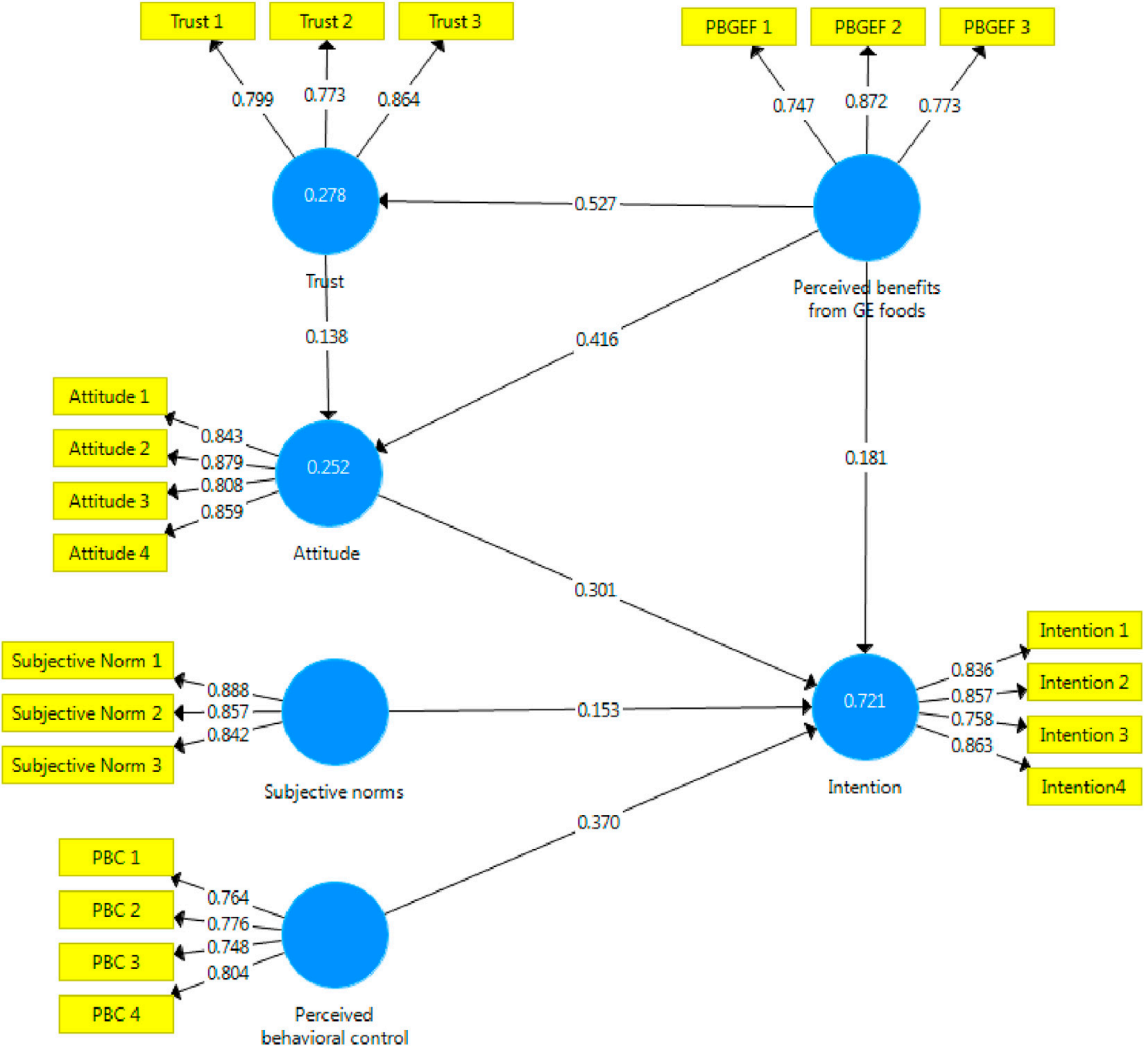
Structural model of the framework.

Also, in the testing section of the measurement model for the intention of consumers of gene-edited products, an attempt was made to examine and analyze the outer weights (Table 5; Figure 2). Outer weights provide the researcher with a criterion to judge the relative weight or importance of each item in the formation of the latent variable. These weights fluctuate between zero and one for different items. The closer an item’s outer weight is to one, the more weighted this item is, and therefore, it can play a more key role in explaining that latent variable. Table 6 shows the weight of each of the items of the latent variables of intention to purchase gene-edited products, attitude towards gene-edited products, perceived behavioral control, subjective norm, trust in gene-edited products, and perceived benefits from GE foods. The results of this section show that all the items mentioned for each of the variables are very important in measuring their corresponding latent variable (Table 5; Figure 2).

**TABLE 6:** Results of evaluating discriminant validity using Fornell-Larcker Criterion.

Variable	Attitude	Intention	Perceived behavioral control	Perceived benefits from GE foods	Subjective norms	Trust
Attitude	0.848					
Intention	0.760	0.830				
Perceived behavioral control	0.759	0.792	0.773			
Perceived benefits from GE foods	0.488	0.556	0.483	0.799		
Subjective norms	0.587	0.647	0.698	0.327	0.863	
Trust	0.357	0.512	0.548	0.527	0.412	0.813

Another part of the results of the evaluation of the measurement model was related to the investigation of construct reliability and validity indicators. Cronbach’s alpha value analysis of the variables of trust in gene-edited products, subjective norms of gene-edited products, perceived benefits from GE foods, perceived benefits from GE foods, intention to purchase gene-edited products and attitude towards gene-edited products showed that all of them have an acceptable Cronbach’s alpha (Table 5). Similarly, the obtained rho_A values of trust in gene-edited products, subjective norms of gene-edited products, perceived benefits from GE foods, intention to purchase gene-edited products and attitude towards gene-edited products were more than the critical value. Composite reliability of all variables was higher than the cut-off value of 0.7. This shows that the Construct reliability and validity statistics were also confirmed.

Finally, the Average Variance Extracted (AVE) of trust in gene-edited products, subjective norms of gene-edited products, perceived benefits from GE foods, intention to purchase gene-edited products and attitude towards gene-edited products was investigated (Table 5). The results of this section showed that the AVE value for these variables were higher than the cutoff value. Therefore, it can be concluded that all criteria of construct reliability and validity were confirmed in the present study (Table 5).

The quality criteria of the model showed that the independent variables were able to explain a significant amount of the variance of the dependent variables in the intention to buy gene-edited products. R Square Adjusted for intention to buy gene-edited products was 0.718. Also, the independent variables were able to explain 0.276 and 0.248 of the variances of trust in gene-edited products and attitude towards gene-edited products, respectively (Figure 2).

Examining the discriminant validity of the variables using the Fornell-Larcker Criterion showed that because the numbers placed in the diameter of the matrix are higher than the values reported in the same column (Table 6). Therefore, the Fornell-Larcker Criterion confirms the discriminant validity in this study (Table 6).

In order to analyze the discriminant validity, another criterion called Cross Loadings was also used, the results of which are given in Table 7. In this discriminant validity evaluation method, each of the items of each variable must have the highest loading on that variable itself. In other words, the loading of each item on its own variable should be higher than that item’s loading on other variables. The results of cross-loadings show that all the items defined for the variables have the highest loadings on their own variables and relatively less loadings on other variables (Table 7). This result shows that in the present study, discriminant validity has been confirmed using cross loadings.

**TABLE 7:** Results of evaluating discriminant validity using cross loadings.

Item	Attitude	Intention	Perceived behavioral control	Perceived benefits from GE foods	Subjective norms	Trust
Attitude 1	0.843	0.768	0.648	0.518	0.546	0.273
Attitude 2	0.879	0.583	0.662	0.343	0.459	0.316
Attitude 3	0.808	0.535	0.629	0.228	0.494	0.264
Attitude 4	0.859	0.637	0.636	0.493	0.480	0.353
Intention 1	0.702	0.836	0.714	0.366	0.586	0.343
Intention 2	0.587	0.857	0.671	0.565	0.508	0.547
Intention 3	0.568	0.758	0.569	0.419	0.543	0.433
Intention 4	0.657	0.863	0.667	0.497	0.511	0.384
PBC 1	0.544	0.664	0.764	0.444	0.571	0.541
PBC 2	0.648	0.551	0.776	0.380	0.510	0.417
PBC 3	0.560	0.526	0.748	0.275	0.529	0.409
PBC 4	0.604	0.683	0.804	0.377	0.544	0.328
PBGEF 1	0.315	0.409	0.351	0.747	0.181	0.469
PBGEF 2	0.485	0.531	0.448	0.872	0.368	0.437
PBGEF 3	0.353	0.376	0.348	0.773	0.212	0.354
Subjective Norm 1	0.476	0.591	0.655	0.260	0.888	0.400
Subjective Norm 2	0.483	0.483	0.514	0.168	0.857	0.188
Subjective Norm 3	0.555	0.587	0.621	0.399	0.842	0.449
Trust 1	0.309	0.497	0.467	0.415	0.469	0.799
Trust 2	0.126	0.285	0.339	0.378	0.157	0.773
Trust 3	0.382	0.438	0.501	0.479	0.339	0.864

The inner variance inflation factors (VIF) showed that there was no VIF above 5; no significant multicollinearity issues were present among the constructs. Attitude toward gene edited products had VIF of 2.514 with intention and 1.1384 with perceived benefits from GE foods and trust. Perceived behavioral control had a VIF of 3.185 with intention. Perceived benefits from GE foods had VIF of 1.384 with attitude, 1.369 with intention, and 1.000 with trust. Subjective norms had a VIF of 1.983 with intention and trust had a VIF of 1.384 with attitude. Overall, there is negligible variance inflation among all variables of trust, subjective norms, perceived benefits, intention and attitude toward gene edited products. The analysis of goodness of fit indicators shows that the research framework has a good fit (Table 8). dULS and dG were significant at the 0.05 error level. This result shows that the estimation of the model has been done effectively. SRMR was 0.165, which shows that the measurement error in the correlation matrix is acceptable. RMStheta cut-off value was 0.12 higher. Therefore, the desired model is a well-specified model (Henseler et al., 2014) (Table 8).

**TABLE 8:** Fit Summary of the model.

Fit index	Saturated model	Estimated model
Standardized Root Mean Square Residual (SRMR)	0.089	0.165
the squared Euclidean distance (d_ULS)	1.840	6.279
the geodesic distance (d_G)	0.842	1.088
Chi-Square	1,563.068	1,770.813
NFI	0.673	0.630
the root mean square error correlation (rms) Theta	-	0.188

## 5 Discussion and recommendations

The results of this study showed that the attitude towards gene-edited food products has a positive and significant effect on the intention to buy these food products. This result is in line with the findings of McFadden et al. (2023), Ploll et al. (2023), Sleboda and Lagerkvist (2022), Ufer et al. (2019), and Ufer et al. (2022). The attitude towards gene-edited food products is considered to mean the favorable or unfavorable orientation of consumers towards these food products. Because in this study, the attitude towards gene-edited food products was conceptualized and operationalized as a positive orientation of consumers, it can be concluded that strengthening consumers’ attitudes towards gene-edited food products can lead to their greater intention to purchase gene-edited food products. This optimistic outlook may stem from a heightened understanding and knowledge of biotechnology advancements in Iran, as well as a growing trust in scientific institutions. In addition, cultural values that focus on good food and secure food supply frameworks may spurn consumers to embrace gene-edited foods. Likewise, there is doubt and ethical scenarios in some consumer segments that may warrant hesitancy on the consumer part, and create a space for sharpe-consumption educational campaigns. It is suggested that in order to improve the attitude towards gene-edited food products and influence the consumers behavioral intention to purchase gene-edited food products by using it (attitude), the current attitudes of consumers be evaluated first. In other words, at this stage, behavioral interventionists should try to identify the negative feelings that consumers have in the context of gene-edited food products and eliminate these negative feelings. In this case, consumers will have a positive attitude towards gene-edited food products, and as a result, their intention to buy these products will be strengthened. Another very practical method to strengthen consumers’ attitude towards gene-edited food products is to refer them to knowledgeable and positive people. In other words, manufacturers and sellers of gene-edited food products should try to make consumers share their uncertainties and sometimes negative feelings with experts. This recommendation means that consumers will not rely solely on what they hear from ignorant people by linking to experts and positive people about gene-edited food products, and this can improve their attitude.

Also, the results of the research showed that the subjective norm of gene-edited food products had a positive and significant effect on consumers’ intention to buy these food products. In other words, the greater the social pressure to buy gene-edited food products, the greater the intention of consumers to buy these products. Similar results have been reported by other researchers including Cummings and Peters (2022), Matsuo and Tachikawa (2022), Nkott and Temple (2021), and Ploll et al. (2023). It can be understood that the subjective norms of buying gene-edited food products is a key determinant of the intention to buy these food products. This influence can be considered in terms of the cultural and social context of Iranian consumers, especially in regard to family ties and social approval. Individual decision making in Iranian society will be impacted by such norms. The culture in Iran is similar to a lot of collectivist cultures around the world, meaning family, friends, and community leaders will strongly affect Iranian consumers in regards to their purchasing behaviors. Furthermore, social media and celebrity endorsements are the next generational avenues reinforcing subjective norms - thus increasing the influence on consumer intention. It is suggested that in order to strengthen the subjective norms of purchasing gene-edited food products, the behaviors of people who play a key role in the identification of consumers and buyers should be highlighted for them. For example, some famous people or celebrities are among the people from whom some consumers take a part of their identity. Therefore, getting to know their perspectives and behavior towards gene-edited food products can create a conscious or unconscious social pressure among the respondents to buy gene-edited food products. Also, family members of consumers of gene-edited food products and even their friends and acquaintances can create subjective norms and positive social pressures towards buying and consuming gene-edited food products. Because it is not possible to communicate with all the acquaintances of the buyers of gene-edited food products for the producers and sellers of these products, advertising the use of these products in mass media such as radio and television and even social networks is one of the useful solutions to create positive subjective norms towards gene-edited food products. This can ultimately strengthen consumers’ intention to buy gene-edited food products.

Perceived behavioral control in the field of buying and using gene-edited food products had a positive and significant effect on the intention to buy these food products. Findings of Araki and Ishii (2015), Bechtold (2018) and Spok et al. (2022) support this result. This variable includes the consumer’s perception of his/her ability to buy gene-edited food products and also his/her control over the situation. Perceived behavioral control is a combination of locus of control (persons’ beliefs about their level of control over their life events and consequences) and self-efficacy (a person’s perceived ability to perform a task). The direct positive effects of perceived behavioral control can likely be attributed to the consumers’ convenience and affordability needs, all key factors in the decision-making process. In the Iranian market context, not only do the consumers have a heightened price sensitivity as they may not experience viable alternatives or substitutes in the marketplace, spending power is also a significant concern. Thus, consumers’ perceptions of themselves having sufficient financial resources suppliers as well as easy access to purchasable gene-edited food products shapes their intention to buy. Also of importance is consumers’ exposure to gene-edited food products in retail choices that are recognized and accessible. Consumer perception of perceived behavioral control also increases when consumers are familiar with the retail outlet space to purchase gene-edited foods, which ultimately builds perceived behavioral control. Therefore, to increase the intention of consumers to buy gene-edited food products using perceived behavioral control, it is suggested that these products be offered at a wider level of sales and distribution centers. This will increase the ease of access or convenience for consumers, and as a result, their intention to buy gene-edited food products will be strengthened. Also, it should be noted that monetary barriers have always acted as a limiting factor for the purchase of many food items such gene-edited food products. Many of consumers will not be able to buy it, so it is recommended that the price of these products be equal to or even lower in comparison with other competing products. It can strengthen perceived behavioral control in the context of purchasing gene-edited food products.

In this study, TPB was developed by adding some variables such as trust in gene-edited food products. Interestingly, this variable had a positive and significant effect on consumers’ intention and attitude towards gene-edited food products. Trust has always been a determining factor for the acceptance of technology or products produced with new technologies such as gene editing. The results of this study emphasize on strengthening the trust of buyers and consumers of gene-edited food products. Such trust may derive from consumers’ perspectives regarding the safety, clarity, and credibility of both the technology used and the institutions involved in producing gene-edited foods. In the case of Iranian consumers, the additional layer of skepticism towards emerging technologies due to limited public information or experience makes the need for trust pivotal. Consumers often depend on clear and honest communication and visible-benefits to encourage them to adopt new products. To strengthen the trust of buyers and consumers of gene-edited food products, perhaps focusing on the benefits of gene editing and gene-edited food products such as Lassoued et al. (2019) which consumers pay attention to is one of the best strategies. Also, to strengthen the trust of consumers and buyers of gene-edited food products, it is recommended to explain to them how these products are produced. Many consumers today do not want to be treated like ignorant people who do not like to understand. In other words, manufacturers and sellers of gene-edited food products should not think of consumers as strangers and untrustworthy people who should not know anything about the production process of the products. Applying these strategies and suggestions can strengthen the trust and ultimately the intention of consumers and buyers of gene-edited food products. In addition, the use of these strategies can indirectly strengthen the intention of buyers and consumers of gene-edited food products due to the effect of trust on attitude.

Perceived benefits of gene-edited food products was also one of the new variables introduced in the TPB. The results of the present study showed that this variable has a significant positive effect on the intention to buy gene-edited food products. This result has been supported by Hendricks et al. (2022), Macall et al. (2023), McFadden et al. (2023), Muringai et al. (2020), and Sleboda and LagerKvist (2022). This important influence is a result of the consumers’ awareness of concrete benefits, such as better nutrition, increased food security, environmental sustainability, and possible cost savings. In the case of Iran, where food safety and quality are incredibly salient concerns, highlighting the perceived benefits will help to lessen the fears surrounding gene-edited foods and cultivate positive perceptions. Moreover, the potential to leverage education to separate gene editing from conventional genetic modification can reduce misunderstandings and increase acceptance. One of the effective strategies in this field is increasing the awareness of consumers about the nature of the gene editing process, the difference between gene editing and genetic modification, the production process of these products, benefits, etc. Producers and providers of gene-edited products can start this awareness program before the production of their products and even after the production of their products and at the time of release on the market, continue with explanations on the product.

There were several major limitations to this study. One of the most important limitations in this study was that due to economic constraints, it was not possible to conduct this study in the whole of Iran. Based on this, it is suggested that future researchers, if they have sufficient financial support, conduct this research in the whole of Iran or even in the other countries. The second limitation was related to the wide range of variables that could affect consumers’ preference for gene-edited food products, but it was not possible to examine all of them in this study. In this regard, it is recommended that researchers use another social, psychological, and even economic variable in future research on the determinants of willingness to buy gene-edited food products.

## 6 Conclusion

This research examined the determinants of intention to purchase gene-edited food products by Iranian consumers with the help of an extended Theory of Planned Behavior (TPB). The main constructs of the TPB, namely, attitudes, subjective norms, and perceived behavioral control, had significant and positive impacts on purchase intention. These results confirm the appropriateness of using the TPB in understanding consumer behavior related to new food technologies and suggest consideration of both individual and social aspects when evaluating purchasing behavior.

Attitude was found to be a significant variable, indicating that when a consumer has a positive attitude towards gene-edited food products, they are more likely to show a willingness to purchase. This supports that affective and cognitive evaluations, like perceived usefulness, safety and alignment to personal values, are most influential in consumer decision-making. A positive attitude toward the technology significantly increased behavioral intention. Subjective norms appeared to also play an important role, especially within a collectivistic culture, like Iran, where purchasing behavior is influenced by friends, family, perceived norms and societal authorities. The significant effect of perceived behavioral control suggests that consumer intentions to purchase these products are impacted by their perceived or actual access and ability to purchase the products. This finding suggests that, in addition to motivation, access—actual or perceived—also promotes behavior.

The new constructs—trust and perceived benefits—provided an even greater amount of explanatory power than TPB on its own. Trust in the safety, transparency, and institutes promoting gene-edited food products was crucial in shaping attitudes and intentions. Perceived benefits, such as improvements in nutrition or the environmentally beneficial features of the food, also enhanced consumers’ willingness to adopt them. Overall, the research results provide a more holistic understanding of the psychological, social, and practical elements affecting consumer acceptance of gene-edited food products in Iran.

## Data Availability

The raw data supporting the conclusions of this article will be made available by the authors, without undue reservation.
