# Supplementary material for: The decision to purchase genome edited food products by Iranian consumers: theory of planned behavior as a social intervention tool
Source: Front Genome Ed. 2025 Sep 4;7:1483510. doi: 10.3389/fgeed.2025.1483510 (PMC12443678; doi:10.3389/fgeed.2025.1483510)
Supplement: Supplementary file 1 [file Table1.docx]

## In the name of God

## Dear participant, this study is being done to explore the constructs predicting consumers’ intention to buy gene-edited food products. Please indicate your level of agreement with each statement on a scale from 1 (Completely Disagree) to 5 (Completely Agree).

## Attitude

| Question | 1 | 2 | 3 | 4 | 5 |
| --- | --- | --- | --- | --- | --- |
| The application of gene editing for food production is very good. |  |  |  |  |  |
| The application of gene editing for food production is very wise. |  |  |  |  |  |
| I completely agree with using gene editing technology for food production. |  |  |  |  |  |
| I don’t think that using gene editing for food production poses any danger to the environment for me or others. |  |  |  |  |  |

## Subjective Norms

| Question | 1 | 2 | 3 | 4 | 5 |
| --- | --- | --- | --- | --- | --- |
| My family encourages me to buy gene-edited products. |  |  |  |  |  |
| Experts and specialists who influence me think I should buy gene-edited products. |  |  |  |  |  |
| My friends encourage me to buy gene-edited products. |  |  |  |  |  |

## Perceived Behavioral Control

| Question | 1 | 2 | 3 | 4 | 5 |
| --- | --- | --- | --- | --- | --- |
| Whether I ultimately buy gene-edited products depends entirely on me. |  |  |  |  |  |
| If gene-edited products are available in the hypermarket, nothing will prevent me from buying them. |  |  |  |  |  |
| Whenever I want, I can easily buy gene-edited products. |  |  |  |  |  |
| I think all consumers have easy access to gene-edited products. |  |  |  |  |  |

## Trust

| Question | 1 | 2 | 3 | 4 | 5 |
| --- | --- | --- | --- | --- | --- |
| I trust gene-edited food products because I’m sure scientists have sufficiently researched their safety. |  |  |  |  |  |
| I trust gene-edited products because I believe the food industry follows strict standards. |  |  |  |  |  |
| I trust gene-edited products because I know decision-makers and policymakers also assure their safety. |  |  |  |  |  |

## Intention

| Question | 1 | 2 | 3 | 4 | 5 |
| --- | --- | --- | --- | --- | --- |
| If gene-edited products are available in the hypermarket, I am willing to buy them. |  |  |  |  |  |
| I recommend buying gene-edited food products to others. |  |  |  |  |  |
| If I don’t find gene-edited products in this hypermarket, I will go to other hypermarkets. |  |  |  |  |  |
| I am willing to buy gene-edited products even if their sale is banned later. |  |  |  |  |  |

## Perceived Benefits

| Question | 1 | 2 | 3 | 4 | 5 |
| --- | --- | --- | --- | --- | --- |
| Gene-edited products have higher nutritional value compared to similar non-edited products. |  |  |  |  |  |
| Gene-edited products generally have relatively lower prices compared to similar products. |  |  |  |  |  |
| Gene-edited products do not pose any health risks to humans. |  |  |  |  |  |

## Demographic Questions

Age: ______

Gender: ( ) Male ( ) Female ( ) Other / Prefer not to say

Education level: ( ) High school or less ( ) Some college ( ) Bachelor’s degree ( ) Graduate degree or higher

Occupation: ____________________

Have you ever purchased gene-edited food products before? ( ) Yes ( ) No

How often do you shop for food products? ( ) Daily ( ) Weekly ( ) Monthly ( ) Rarely
